# Hausdorff Distance Model-Based Identity Authentication for IP Circuits in Service-Centric Internet-of-Things Environment†

**DOI:** 10.3390/s19030487

**Published:** 2019-01-24

**Authors:** Wei Liang, Weihong Huang, Wuhui Chen, Kuan-Ching Li, Keqin Li

**Affiliations:** 1 School of Opto-Electronic and Communication Engineering, Xiamen University of Technology, Xiamen 361024, China; wliang@xmut.edu.cn; 2 Key Laboratory of Fujian University of Internet of Things Applied Technology, Xiamen University of Technology, Xiamen 361024, China; 3 College of Computer Science and Electronic Engineering, Hunan University, Changsha 410082, China; whhuang@hnu.edu.cn; 4 School of Data and Computer Science, Sun Yat-Sen University, Guangzhou 510275, China; chenwuh@mail.sysu.edu.cn; 5 Department of Computer Science and Information Engineering, Providence University, Taichung 43301, Taiwan; 6 Department of Computer Science, State University of New York New Paltz, New York 12561, USA; lik@newpaltz.edu

**Keywords:** Hausdorff distance model, IP circuit, Internet of Things

## Abstract

Rapid advances in the Internet-of-Things (IoT) have exposed the underlying hardware devices to security threats. As the major component of hardware devices, the integrated circuit (IC) chip also suffers the threat of illegal, malicious attacks. To protect against attacks and vulnerabilities of a chip, a credible authentication is of fundamental importance. In this paper, we propose a Hausdorff distance-based method to authenticate the identity of IC chips in IoT environments, where the structure is analyzed, and the lookup table (LUT) resources are treated as a set of reconfigurable nodes in field programmable gate array (FPGA)-based IC design. Unused LUT resources are selected for insertion of the copyright information by using the depth-first search algorithm, and the random positions are reordered with the Hausdorff distance matching function next, so these positions are mapped to satisfy the specific constraints of the optimal watermark positions. If the authentication process is activated, virtual positions are mapped to the initial key file, yet the identity of the IC designed can be authenticated using the mapping relationship of the Hausdorff distance function. Experimental results show that the proposed method achieves good randomness and secrecy in watermark embedding, as well the extra resource overhead caused by watermarks are promising.

## 1. Introduction

In recent years, with the widespread use and development of the Internet of Things (IoT), security issues on hardware have gained attention and wide concern [1,2]. With intellectual property (IP) as the primary module in designing a complex system-on-chip (SoC), the reuse of IP can significantly reduce design and prototyping costs, shortening also the design cycle. Notwithstanding this, IP infringements frequently occur with the massive increase in the usage and quantity of devices and, thus, security primitives are implemented with increasing frequency for protection. For example, Castillo et al. [3] utilized the lookup tables (LUTs) for watermark embedding, where the copyright data is inserted into the space between the used and unused LUTs, and attackers are tough enough to remove since it is concealed into the functional resources of the design. Therefore, the authentication requires an extraction circuit to extract the secret data, causing substantial hardware overhead, and the redundant extraction circuit can also easily be attacked. Chen et al. [4] proposed a public IP authentication scheme, where a pseudo-random identity data is encrypted and inserted into the original design as specific constraints, due to its public capability to satisfy the security requirements and can be detected by graph coloring and Boolean satisfiability proof. Further research used the unused port of the used LUT for watermark insertion [5,6,7,8,9], so the watermarked LUTs are transformed into Random Access Memory (RAM) or Linear Feedback Shift Register (LFSR) for enhancing the ability against the removal attacks. If any infringement occurs, the copyright information can be detected from the bitfile of the design. 

Nevertheless, the above methods easily leak the real positions of the watermarks, which exposes the copyright authentication to threat. To address this issue, Saha et al. [10] proposed to authenticate the IP identity by using a zero-knowledge proof protocol. Not leaking the really sensitive information, such as the watermark position or the watermarked content [11], can enable it to resist tampering attacks, but unfortunately the ability against removal attacks is also lower. If the watermark is impaired after being attacked, the copyright authentication may fail. Zhang et al. [12] used an obfuscation technique to enhance hardware security. 

**Figure 1:**
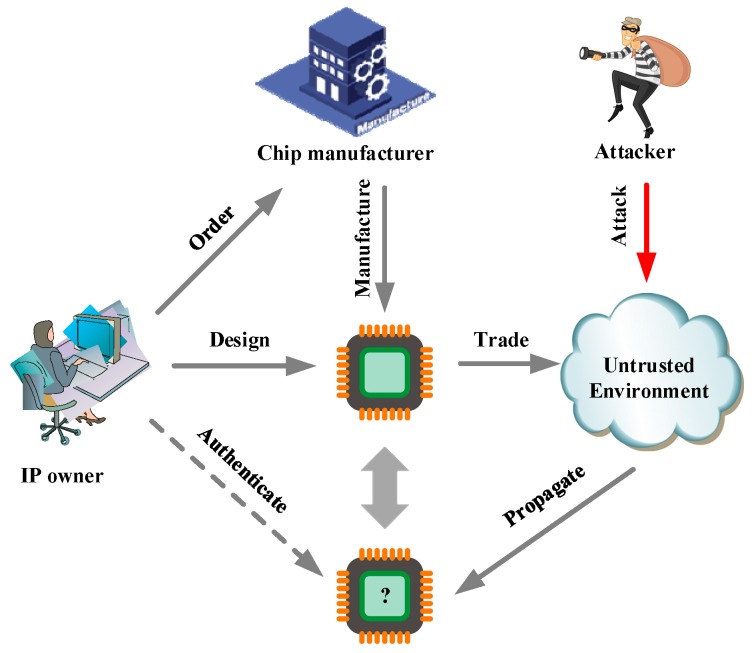
The structure of IP authentication system.

Some IP protection techniques are based on the encrypted bitfile, which requires extra decryption logic, and may cause an increase of the hardware resource and power consumption; for instance, the bitfile of a design implemented on a Static Ramdom Access Memory (SRAM) based field programmable gate array (FPGA) is stored in the external memory, e.g., Electrically Erasable Programmable Read—Only Memory (EEPROM). As the FPGA powers up, the bitstream is loaded into the FPGA device [13]. Depicted in Figure 1, the IP owner designed an IP core that propagates in an untrusted environment. In this case, the IP core may be attacked by illegal attackers. If the infringement occurs, the IP owner can authenticate the identity of the IP.

In recent years, many IP protection methods have been proposed to deter the infringement behavior [14,15,16,17,18,19,20] and have shown good performance to address security issues in IP protection. Nevertheless, there are risks. The misappropriation of the kernel IP module causes significant economic loss to IP owners and developers.

In this work, a robust and secure authentication method is designed and proposed to authenticate the copyright of IP circuit chips in IoT environments. The Hausdorff distance shows excellent performance in fault tolerance and the ability of anti-interference, which is suitable for IP authentication. The theory of Hausdorff distance is introduced first and then an IP protection method proposed for the designed Hausdorff distance model, by which we could realize authentication using the non-similarity of two point-sets, M and N. If the isolated points in M are far away from N and also the matching degree of two sets is small, the larger values of unidirectional and bidirectional Hausdorff distances increase the similarity degree of two point-sets. The similarity between two IP designs is addressed by using the average Hausdorff distance, as the average Hausdorff distance considers the contribution from every point during the calculation of the Hausdorff distance. 

The remainder of this paper is organized as follows. The constraint model based on Hausdorff distance is designed and described in Section 2, and the precise technique introduced for IP copyright authentication is presented in Section 3, where we concentrate on designing virtual node positions for inserting identification information. Experimental results are evaluated and compared to those from other techniques in Section 4, and finally, the paper is summarized as well as some future directions are included in Section 5.

## 2. Preliminaries

Digital IP circuit copyright authentication is a technique to securely protect the ownership of IP design. The IP designer inserts the copyright information in the circuit, which can indicate the identity of the designed IP cores. The inserted content of the copyright information is the binary information after encoding. When IP disputes occur, the designer can authenticate the original identity of the IP by extracting the inserted information in the circuit. In this work, a Hausdorff distance-based constraint function is proposed. The watermarks can be inserted into specific positions after virtual mapping. Within an accepted error range ε, the reverse method of mapping can be used to find the LUT resources which are relevant to the copyright information. 

### 2.1. Intellectual Property (IP) Circuit Based on Hausdorff Distance

**Figure 2:**
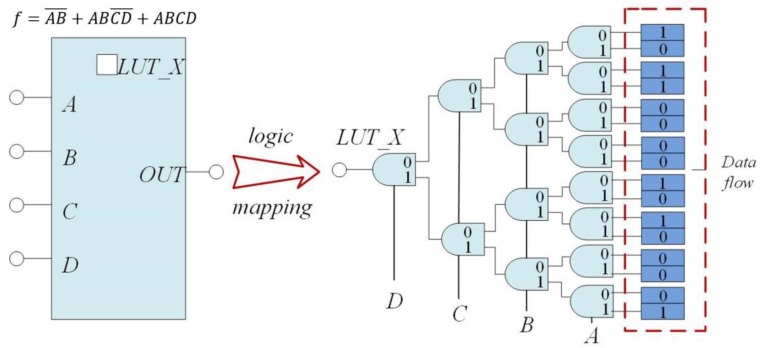
The structure of a lookup table (LUT) configured as a function $f=\bar{AB}+AB\bar{CD}+ABCD$.

There are different types of IP cores at various design levels, as identification information can be inserted at any design level as a part of the IP core for protection [21,22]. FPGAs are programmable integrated circuits, and the process to insert identification information in such a circuit as a target is more complicated than that of traditional multimedia—text, software, among others. There are lots of configurable logic blocks (CLB) in an FPGA device, as each CLB includes several slices and some inner connections. LUTs in each slice can be configured as specific logic functions, and as a LUT is unused, we can insert specific data without affecting the performance of a design so that it can be used as the carrier of a watermark. Figure 2 shows the LUT configured as a logic function $f=\bar{AB}+AB\bar{CD}+ABCD$. With a specific input within 0~15 range, the corresponding result of the function is outputted. 

Kahng et al. [23] proposed to hide the identification data into LUTs of FPGAs, as LUTs are ideal carriers due to the large number of LUTs in an FPGA-based IP core. A LUT can be treated as a RAM where data is written, as input generates an address to search the corresponding content which is the output. The recently used LUTs inter-relate to original LUTs with some “don’t care” connections to enhance the security. In [24], the identification data is inserted into the STG (state transition graph) to authenticate the design copyright, which can be applied to the sequential design of a firm core. Such a technique inserts identification data in FSM (finite state machine) of IP design, which improves the robustness of the watermarks and can be safely implemented [25]. This was the first public IP watermarking technique developed at FSM level. Later, constraint-based watermarking techniques brought additional constraints into a watermarked design [26], which brought extra overheads and degraded the performance.

Notwithstanding this, the constraints added at higher level caused some unpredictable issues [27]. The primary purpose of IP protection methods is to prevent IP copyright in chips from being inappropriately accessed. In practice, the authentication system is implemented with passwords and key encryption, whose security is insufficient. Malicious attackers can perform deep attacks on the electronic chip, such as cloning or collusion attacks. If the chip has higher requirements on the security level, such a system cannot meet the demands [28]. 

To strengthen the security of IP authentication, Xu et al. [29] proposed a chaos mapping-based IP protection method with better reliability and security. However, this also leaks sensitive information of IP watermarks during authentication, such as position or content. Additionally, the constrained watermarks cannot be detected at another design level, where the low traceability is also a drawback. Cui et al. proposed an ultra-low overhead dynamic watermarking on scan design for hard IP protection [30], which has greatly improved the security of the watermarks and reduced the overhead.

**Figure 3:**
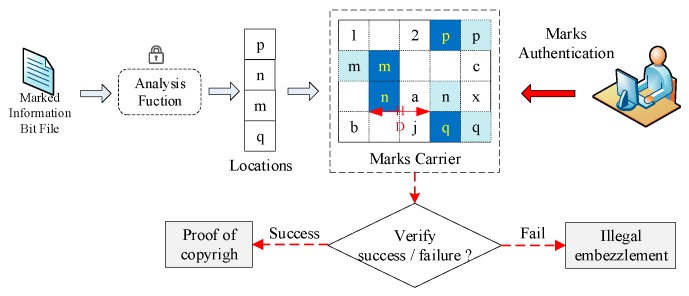
IP authentication based on the Hausdorff distance model.

The Hausdorff distance-based position selection algorithm is to search the unused LUTs around the original design and utilize them in inserting the identification data. Due to serious IP infringements in chip design, researchers exhibit high demands on IP identification techniques. As a popular IP protection technique, IP identification can provide copyright authentication and avoid IP infringement. In this work, random positions around the original design are utilized for identification yet provide real-time protection for IP cores, and the major advantage is the insertion of an enormous amount of identification data. Once attacked, the identification data can also be recovered with the created mapping relationship. Figure 3 shows the diagram of the Hausdorff distance-based IP authentication technique, whereas the IP owner inserts the processed ownership information into the bitfile core. Each IP design contains the information of different users, which is helpful support for tracing the infringement. Such an IP owner can authenticate the IP design with the Hausdorff Distance-based technique. If the ownership information is successfully detected, the copyright is proven; otherwise, the IP design is embezzled. 

### 2.2. Hausdorff Distance-Based Constraint Model

Hausdorff distance (HD) originated from the differential dynamic [31,32] that describes the similarity between two sets of points and widely used in fields of secure identification, since it requires calculating point-to-point distances. In this work, we define the maximum and minimum distances between two sets of IP position points. The maximum mismatching degree between two sets of the point is calculated, so a smaller HD value signifies that the sets are similar; otherwise, the sets have lower similarity. The definitions are as follows.

**Definition** **1**. *Given two sets of point A={a_1_, a_2_, a_3_, …, a_p_} and B={b_1_, b_2_, b_3_, …, b_q_}, the Hausdorff Distance-based constraint between A and B is defined as follows*.

(1) H(A,B)=max(h(A,B),h(B,A))

(2) $$h(A,B)=\underset{a\in A}{\max}\;\underset{b\in B}{\min}\left\|a-b\right\|$$

(3) $$h(B,A)=\underset{b\in B}{\max}\;\underset{a\in A}{\min}\left\|b-a\right\|$$

As in (2) and (3), *h*(*A,B*) is the directed Hausdorff distance from *A* to *B*, and *h(B,A)* is the directed Hausdorff distance from *B* to *A*, with ║║as the norm distance of *A* and *B*, *h(A,B)* is seen as the maximum value of distances of each point in *A* to *B*, and *h(B,A)* the maximum distance value of points in *B* to *A*. From this, HD is obtained from the maximum mismatching degree between *A* and *B* by calculating the maximum value of *h(A,B)* and *h(B,A)*.

**Definition** **2**. *If A and B satisfy affine transformation relationship, HF_a_(x_a_,y_b_) and HF_b_(x_b_,y_b_) are two points respectively in A and B, the mapping relationship is defined as (4)*:

(4) $$\left\{ \begin{array}{l} HF_{a}=m_{00}x_{a}+m_{01}y_{a}+l_{x}\\ HF_{b}=m_{10}x_{a}+m_{11}y_{a}+l_{y} \end{array}\right.$$

Here, *l_x_* and *l_y_* are, respectively, the offset in *x* and *y* directions, and the vector HF_ab_=(*m*_00_,*m*_01_,*m*_10_,*m*_11_,*l_x_*,*l_y_*) denotes the matching relationship. Based on Definition 1, the transformation of Hausdorff distance at two sets of points in LUTs is defined as (5), deriving (6) and (7).

(5) $$H_{LK}\left(A,B\right)=\max\left(h_{L}\left(A,B\right),h_{K}\left(B,A\right)\right)$$

(6) $$h_{L}\left(A,B\right)=L_{a_{i}\in A}^{th}\underset{b_{j}\in B}{\min}\left\|a_{i}-b_{j}\right\|$$

(7) $$h_{K}\left(B,A\right)=L_{b_{j}\in B}^{th}\underset{a_{i}\in A}{\min}\left\|b_{j}-a_{i}\right\|$$

In Equation (6), $L_{a_{i}\in A}^{th}$ represents the *L*-th value of the unidirectional Hausdorff distances from *A* to *B* in descending order, $K_{b_{j}\in B}^{th}$ the *K*-th value of the unidirectional Hausdorff distances from *B* to *A* in descending order. As *K* = *L* = 1, Equation (5) is the original Hausdorff distance from *A* to *B*. To eliminate the interference of isolated points, the sensitivity of Hausdorff distance is reduced. However, there exists the case that HD cannot accurately describe the similarity between point sets. Thus, average Hausdorff distance is applied in (9) to address issues of similar type.

(8) $$H_{mean}\left(A,B\right)=\max\left(h_{mean}\left(A,B\right),h_{mean}\left(B,A\right)\right)$$

Here, we have:

(9) $$h_{mean}\left(A,B\right)=\frac{1}{N_{A}}\sum_{a_{i}\in A}\left(\underset{b_{j}\in B}{\min}\left\|a_{i}-b_{j}\right\|\right)$$

(10) $$h_{mean}\left(B,A\right)=\frac{1}{N_{B}}\sum_{b_{j}\in B}\left(\underset{a_{i}\in A}{\min}\left\|b_{j}-a_{i}\right\|\right)$$

In Equation (9), *N_A_* is the number of characteristic points in *A*, and *N_B_* in (10) the number of characteristic points in *B*. The average Hausdorff distance considers the contribution of each point in calculating the Hausdorff distance and, therefore, it is real and of higher accuracy than the description of the similarity degree between two points of the set.

(11) $$H\left(t_{affine}\left[A\right],B\right)=\max\left(h\left(t_{affine}\left[A\right],B\right),h\left(B,t_{affine}\left[A\right]\right)\right)$$

The Hausdorff distance coefficient represents the matching degree between the positions of identification data and the constraint function. In the case with lower requirements for accuracy, it can reduce the computation complexity without affecting the performance by setting the small component of Hausdorff distance coefficient to zero. Based on this fact, the component of the Hausdorff distance coefficient with the most considerable absolute value will be regarded as the position constraint characteristic of the IP circuit, and the high-frequency coefficient in the position constraint function of IP circuit is set to zero. After that, it will be reconfigured. The maximum absolute value of the Hausdorff distance coefficient is calculated as the candidate position characteristic vector. With principal component analysis (PCA) and normalization processing, the position characteristic vector between two points is obtained, so the collection of position characteristic vectors is successfully extracted.

Assuming that the response signal of the IP circuit is resolved by the position constraint function, the *a*-th component of the *b*-th Hausdorff distance coefficient is denoted by $h_{a}^{b}\;\left(a=1,2,3,\;\ldots ,\;N\right)$. Consequently, the candidate position characteristic *H_b_* is defined as (12).

(12) $$H_{b}=\max\left(\left|h_{a}^{b}\right|\right),a=1,2,\cdots ,M$$

where *M* is the dimension of the Hausdorff distance coefficient. The candidate position characteristic vector is denoted as (13) and (14).

(13) $$HW_{x}=\varepsilon {\left[x_{1},x_{2},\cdots ,x_{a},\cdots x_{N}\right]}^{T}$$

(14) $$HW_{y}=\varepsilon {\left[y_{1},y_{2},\cdots ,y_{b},\cdots y_{N}\right]}^{T}$$

The position searching function *HS* can be used to search exact positions of related points, and is represented as follows:

(15) $$HS_{x}=\min H_{ab}\left(U,V,W\right)=\sum_{a=1}^{N}\sum_{b=1}^{N}u_{ab}^{m}{\left\|x_{b}-x_{a}\right\|}^{2}$$

The constraint is described as (16):

(16) $$\begin{array}{l} \forall a\in \left\{1,2,\cdots ,N\right\},b\in \left\{1,2,\cdots ,N\right\}\\ u_{ab}\in \left[0,1\right],\sum_{a=1}^{N}u_{ab}=1,0<\sum_{b=1}^{N}u_{ab}\leq N \end{array}$$

In (15), *U* is the position collection of LUT array, *V* is the position collection array of the center, *W* is the collection of identification data, *c* is the number of clusters, *x_b_*∈*R_p_* denotes the *b*-th data pattern, and $u_{ab}$ is the position degree of *x_b_* belonging to the *b*-th category.

The position constraint function in (15) is normalized, yielding the position characteristic vector of Hausdorff distance. The normalized position constraint function is described as (17):

(17) $$\begin{array}{ll} HS_{AB} & =\underset{\left(U,V,W\right)}{\min}H_{ab}\left(U,V,W\right)\\ & =\sum_{a=1}^{N}\sum_{b=1}^{N}u_{ab}^{m}{\left\|x_{b}-v_{b}\right\|}^{2}+\frac{\beta }{m^{2}\sqrt{c}}\sum_{a=1}^{N}\sum_{b=1}^{N}\left(u_{ab}^{m}\log u_{ab}^{m}-u_{ab}^{m}\right) \end{array}$$

## 3. Hausdorff Distance-Based Authentication Algorithm

This section introduces the Hausdorff distance-based authentication algorithm, and the symbols used are as follows: H(A,B) is the bidirectional Hausdorff distance, and h(A,B) and h(B,A) are the unidirectional Hausdorff distance from *A* to *B* and *B* to *A* respectively. The distance of two points, *a* in *A* and *b* in *B*, can be calculated by a−b. The calculated distances are ordered to get the maximum one, denoted by h(A,B), and similarly the h(B,A). The value of H(A,B) is the highest value of h(A,B) and h(B,A), denoting the non-similarity degree of two point-sets. Thus, the position constraint function is analyzed with Equation (11). The main steps of IP authentication are illustrated, including position selection, identification data insertion, identification matching, and copyright authentication. 

### 3.1. Position Selection

In this work, a real-time IP authentication algorithm based on the Hausdorff distance constraint model is proposed to insert the identification data into IP circuits effectively. This searches the unused LUTs from the FPGA-based IP design, as well those secure positions analyzed to find out those which cannot be attacked. From (21), it is defined as a position constraint factor ε to constrain the positions of identification data, whose condition is that the similarity of Hausdorff distance represents the difference of IP circuits. 

The design of a position selection algorithm to search suitable positions for inserting identification data is presented next, and the steps are as follows. First, a search to unused LUTs with the length of identification data is processed, and the similarity between the original IP design and the watermarked IP design is calculated by the Hausdorff distance model next. The value of Hausdorff distance is zero if both circuits match to be the same. Though, due to the insertion of the identification data, the value will not be zero. A matching error η exists in the calculation of Hausdorff distance, so it should be reduced to an acceptable range ε. The identification data is recorded in a key file for better security. Besides, the position mapping algorithm is utilized to prevent the key file from being leaked. The Hausdorff distance-based authentication method can determine the mapping relationship between the positions of the original design and the watermarked design. If the matching error falls in the range ε, then the selected positions for identification data insertion are secure; otherwise, the positions should be selected again by using the above steps. The pseudo-code of the algorithm is described in Algorithm 1.

**Algorithm 1**: IP Marks embedding the algorithmInput: (i) Position $P_{n}$ (ii) Marks sequence W
Output: (i) Pseudo position $L_{n}$ (ii) Constraint function $HW_{xy}$
/*Marks sequence $W=\{w_{0},w_{1},w_{2},\ldots ,w_{n}\}$, L means that P after the Marks sequence is embedded, the virtual position generated by the constraint $HW_{xy}$*/ /* $C_{n}$ represent unused resource sequence $C_{n}$*/ 1: Single location information $P_{i}$ selected from $C_{n}$; 2: Calculate $HW_{xy}$ by Constraint function $HF_{ab}$ and $P_{i}$; 3: Calculate Single pseudo location information $L_{i}$ by Constraint function $HW_{xy}$ and $P_{i}$; 4: If $L_{i}\in C_{n}$ then 5. Store location information $P_{i}$ and $L_{i}$; 6: Else if $L_{i}\notin C_{n}$ then 7: Delete the element $P_{i}$ from the collection $P_{n}$; 8: End If 9: Store $P_{i}$ in $P_{n}$; 10: For i:= 0 to n
11: Embed $w_{i}$ into Location $P_{i}$ in $P_{n}$; 12: End For 13: Output $L_{n}$ and $HW_{xy}$;

### 3.2. Position Characteristic Matching

(1) Position selection. LUT resources in FPGA can be represented as a M*N array, and the position of a LUT is given by $\left(x_{a},y_{b}\right)$. The product of area errors can provide the position matching value, and the similarity is expressed by (18).

(18) $$SAD\left(u,v\right)=\iint_{\Phi }\left|f_{1}\left(x,y\right)-f_{2}\left(x+u,y+v\right)\right|dxdy$$

Equation (18) is normalized to generate (19). Hereafter, f_1_ and f_2_ are inserted and extracted identification data, respectively.

(19) $$SAD(u,v)=\iint_{\phi }\left|(f_{1}(x,y)-\bar{f_{1}}\right)-(f_{2}(x+u,y+v)-\bar{f_{2}}(u,v))|dxdy$$

The position characteristic value of the Hausdorff distance is generated after PCA analysis and normalization. With (19), the characteristic matching value SAD is obtained.

HD matching method is to match the IP circuit including the selected positions of identification data with the one that includes the unselected positions of identification data. The most suitable initial positions of identification data are found within an acceptable range ε, denoted by LOC1.

**Figure 4:**
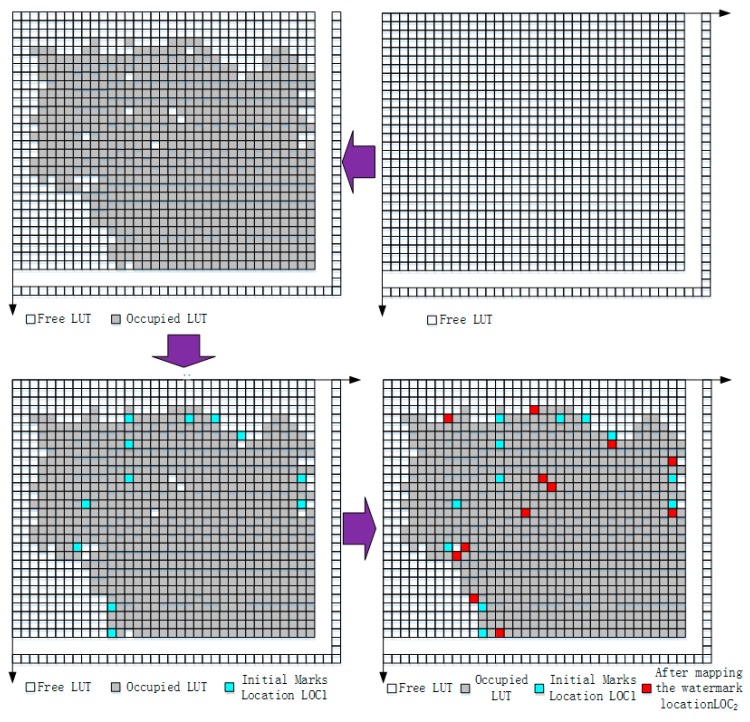
The distribution of identification data.

(2) Position mapping. The positions selected by (1) should be further mapped with Definition 2, and the distribution of the identification data is shown in Figure 4. Selected LUTs with Equation (8) are different from the initially selected positions. The Hausdorff distance matching algorithm is used to verify the mapped positions of the identification data. If the Hausdorff distance falls into the range ε, the set of the mapped positions is denoted by LOC2. Otherwise, the selected positions cannot be used for identification data insertion and should be selected again.

(3) Identification data insertion. Detailed steps of insertion are as follows.

1. Generate the IP design for identification data insertion. The integrated circuit is implemented using design tools such as ISE or Quartus, Modelsim and Synplify synthesis tool for further implementation. The bitfile design is generated for a specific FPGA device.
2. Generate the identification data. The insertion procedure is illustrated by an example of inserting two identification data, M1 and M2, that should be transformed into binary data at first.
3. Search the positions for inserting identification data by traversing the point set and indexes ascendingly. The binary data is orderly inserted into the selected positions. With the traversing algorithm, the characteristic value of the selected position can be calculated. Meanwhile, it can be regarded as the clue to determine the virtual positions, whereas the positions are stored with tree indexes. Besides, the priority queue is used to control the accessed sub-nodes.

- Classifying the positions of LUTs with scanning algorithm, i.e., used and unused LUT collection. In the unused collection, the LUTs with the number equal to the fragments of the identification data are selected. The Hausdorff distance is applied to calculate the characteristic matching degree between the IP cores that includes the selected positions and those with the unselected positions. Within the constraint range ε, a position collection for inserting identification data can be determined and denoted by LOC1. LOC1 is stored in a key file, and the position mapping algorithm is further mapped to get virtual position collection LOC2. In this case, the real positions are covered to ensure better security. Finally, Hausdorff distance is used to verify LOC2, if the verification result is within the range of ε. If positive, the mapping will be successful; the positions should be selected again if otherwise. The position mapping can avoid the attackers obtaining the real positions of identification data from the key file. Thus, the stored position collection LOC1 in the key file is not the real position to insert the identification data.
- Modify or replace the control state of LUT resources. The generated identification data can be inserted into the selected secure positions, as some extra connections are added to make the newly added resources integrate with the functional resources, and potentially enhance the security of the identification data. The identification data matching algorithm is depicted in Algorithm 2.

**Algorithm 2**: IP Marks matching algorithmInput: (i) Pseudo Position $L_{n}$ (ii) Marks sequence $W^{\prime }$
Output: (i) Current design $L_{n}^{\prime }$
/*Marks sequence $W\prime =\{w\prime_{0},w\prime_{1},w\prime_{2},\ldots ,w\prime_{n}\}$, Current design $L_{n}^{\prime }$ means that the location of the information for embedding the Marks */ /*Compare the size of $L_{n}$ and $W^{\prime }$*/ 1: gets($L_{n}$); gets($W^{\prime }$); /*Traversing each element of the collection $L_{n}$ and the collection $W^{\prime }$*/ 2: for (i=0; L[i]!=’\0’&&$W^{\prime }\left[i\right]$!=’\0’; i++); 3: if (L[i]≠W′[i]) 4: break; 5: else if $L\left[i\right]=W^{\prime }\left[i\right]$
6: Embed $W^{\prime }\left[i\right]$ into Location L[i] in $L_{n}$; 7: Store L[i] in $L_{n}^{\prime }$; 8: End If 9: End For 10: Output $L_{n}^{\prime }$;

### 3.3. Authentication of Identification Data

**Figure 5:**
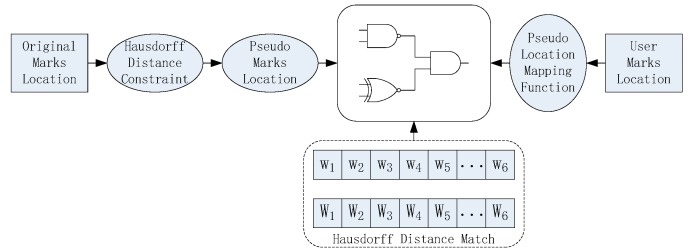
The detailed authentication flow chart.

If IP infringement occurs, the authentication process is activated, and the authentication algorithm is processed in four steps: identification data locating, matching, data combination, and authentication, as shown in Figure 5. 

(1) Identification data extraction. The virtual position set LOC2 can be determined with LOC1 recorded in the key file and the mapping algorithm. For the bitfile design or the physical layout, the fragments of the identification data can be extracted from specific LUTs.

(2) Fragments combination. In step 1, the fragments are extracted and ordered to acquire two binary sequences that include the identification data and the address relation factor. Therefore, the sequences should be split to retrieve the original watermarked positions. Based on the reverse procedure of insertion, the sequence should be divided by the same length to obtain the identification data fragments. These fragments are then combined to get the original positions and the virtual positions.

(3) Decryption and authentication. The stored key can decrypt virtual positions, and after that, the generated binary sequence is also transformed into plain text, as well the decrypted and declared data perform similarity matching operations. If the similarity degree is high enough, it will prove the legality of IP copyright.

The authentication algorithm of identification data is described in pseudo-code depicted in Algorithm 3.

**Algorithm 3**: IP Marks Authentication algorithmInput: (i) Position $P_{n}$ and ${L^{\prime }}_{n}$ (ii) Constraint function $HW_{xy}$
Output: (i) Legal user (ii) Illegal user /* To satisfy the condition $HW_{xy}$, ${L^{\prime }}_{n}$ and $P_{n}$ are compared, and then judge whether ${L^{\prime }}_{n}$ is legal or not */ /* ${L^{\prime \prime }}_{n}$ represents the element that satisfies the condition of $HW_{xy}$,$S_{n}$ represents the same element */ 1: Calculate ${L^{\prime \prime }}_{n}$ according to ${L^{\prime }}_{n}$ and $HW_{xy}$
/* Compare each of $S_{n}$ and $P_{n}$ elements */ 2: gets(${L^{\prime \prime }}_{n}$); gets($P_{n}$); for (i = 0; i < sizeof(${L^{\prime \prime }}_{n}$) / sizeof(${L^{\prime \prime }}_{n}\left[0\right]$); i++) {    for (j = 0; j < sizeof($P_{n}$) / sizeof($P_{n}\left[0\right]$); j++)     {       if (${L^{\prime \prime }}_{n}\left[i\right]$==$P_{n}\left[i\right]$)       {        Store ${L^{\prime \prime }}_{n}$ in $S_{n}$;       }     }   } 3: End for /* Evaluate the similarity */ 4: If (sizeof($S_{n}$) / sizeof($P_{n}$) ≥ 0.8) 5: Output: Legal user 6: else if (sizeof($S_{n}$) / sizeof($P_{n}$) < 0.8) 7: Output: Illegal user;

## 4. Experimental Results and Analysis

We have conducted experiments to evaluate the performance of the proposed method. The IP cores, such as DES, Cache, and RS, are implemented on XC2V800 FPGA device (Xilinx Inc., San Jose, CA, USA), and the tools utilized for evaluation include ISE, Modelsim, and Synplify. The performance evaluations focused on the stability, resource overhead, anti-attack ability, and similarity.

### 4.1. Stability Evaluation

**Figure 6:**
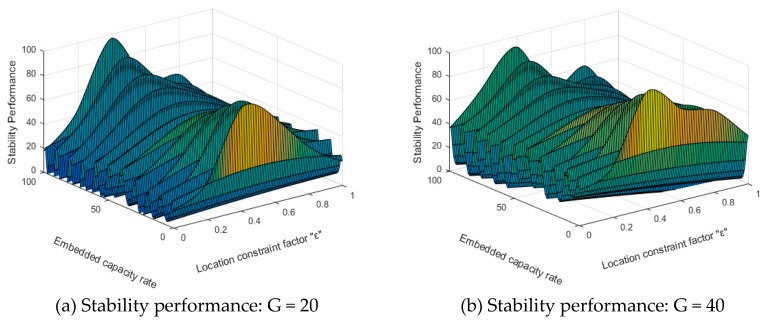
The stability performance evaluation.

Regarding stability, Hausdorff distance is used to determine the virtual positions for identification of data insertion. Under the control of coefficient ε, virtual positions will be distributed among the functional resources. This addresses the issue that attackers can find the real positions of identification data by analyzing attacks. Besides, the initial positions are further mapped to ensure security. The initial positions are stored in the key file, despite the real positions for inserting the identification data are the mapped ones. Even if the attacks retrieve the key file and extract the initial positions, it cannot determine the real positions of identification data. The Hausdorff distance constraint function is used to match the LUTs and make the inserted identification date secure. As the copyright needs authentication, the user can apply for the authentication parameters from the IP owner and perform authentication using the parameters. Therefore, even if the identification data is successfully analyzed and tampered by the attackers, it cannot pass the matching verification. As shown in Figure 6, we set two attack strengths, G = 20 and G = 40, where the stability performance is gentle, demonstrating the validity of the proposed scheme.

### 4.2. Resource Overhead

Extra resource overhead after identification data insertion is evaluated and compared to three methods, as listed in Table 1. Four IP circuits are selected in this experiment, respectively Audio, DES, RS, and Cache. The proposed scheme utilizes the unused resources for identification data insertion, and the occupied resources slightly increase. Herein, “Time” represents the overall time to complete the authentication.

The proposed method can authenticate the copyright in real-time. By contrast with previous works where schemes authenticate the identity of the IP circuit by using multiple rounds of inquiry and the real-time authentication is affected, operations are involved in each round of authentication. In this work, the authentication is based on the Hausdorff distance model, and matching operation based on Hausdorff distance utilized for position calculation. The random positions are ordered and used to realize position mapping, as virtual positions satisfy the constraint condition of the optimal collection of positions. With the authentication activated, the virtual positions in the key file are used and decrypted for copyright authentication. In this section, we evaluate the overhead of the proposed scheme and the comparative schemes [28,29,30]. In [28], the authors proposed a robust low overhead IP watermarking algorithm. It compressed the real watermarks, which greatly reduced the overhead. The use of position mapping can enhance the security of the inserted watermarks. In [29], the authors proposed a high polymetric mutual mapping IP watermarking algorithm. This uses the principle of the secret segmentation mechanism to build a mutual mapping connection between two watermarks. The algorithm can resist the removal attacks. The authors in [30] utilized the structure of scan design and realized an ultra-low overhead watermarking scheme. The result of overhead evaluation is shown in Table 1, and the operation time for the comparative schemes is larger than the proposed schemes. Also, the authentication efficiency is better than similar schemes.

**Figure 7:**
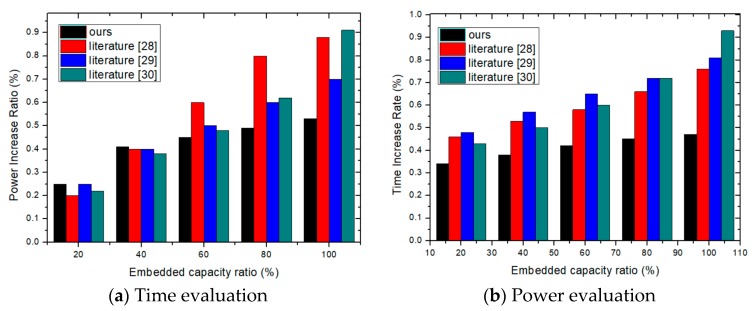
Time and power overhead evaluation with the increase of embedded capacity.

In the proposed scheme, original identification data is encrypted using a hash function that compresses data into a message with the length of 128 bits, and the message is inserted into the design after being divided into a few fragments next. In this experiment, the power and time are evaluated with the increase of inserting identification data from 4 bits to 128 bits. The resource overhead is shown in Figure 7, where Figure 7a shows the time increase rate and Figure 7b the power increase ratio. With the increase of the embedded capacity ratio, the overhead curves of the three schemes are ascending, so the increase in time and power for the proposed scheme is the minimum (indicated as “ours”).

**Figure 8:**
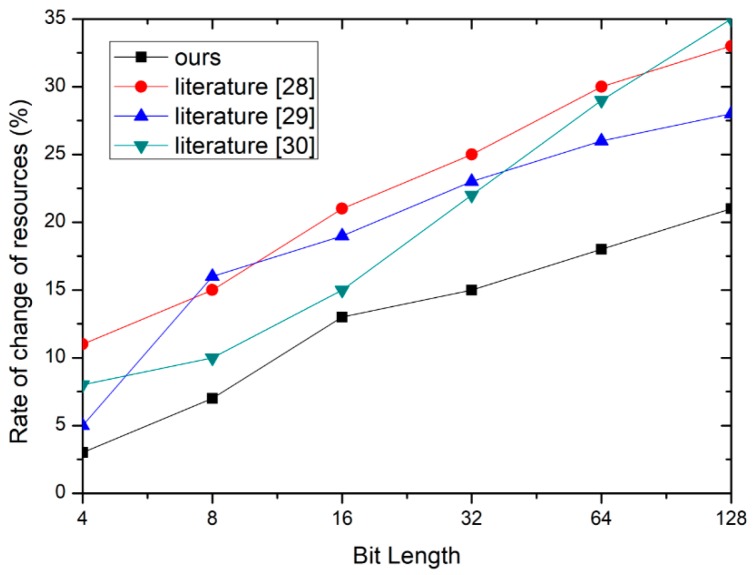
The evaluation of resource overhead.

Figure 8 shows the rate of resource change. From this figure, the increase in resources is the lowest by comparison with the other three methods.

### 4.3. Anti-Attack Ability

**Figure 9:**
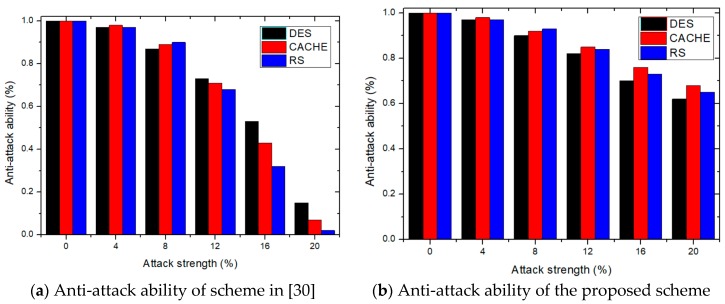
The evaluation and comparison of the anti-attack ability.

Several attacks threaten the security of IP design and IP watermarks. In this section, the replay attack is mainly considered to evaluate the security of position selection. First, the selected IP designs are implemented in the Xilinx ISE tool, with the identification data inserted into the designs of the proposed method. The generated design is the watermarked core, and attacked for performance evaluation purposes. In Figure 9, the X-axis denotes the attack strength, while the Y-axis denotes anti-attack ability. Figure 9a is the anti-attack ability of [30] and Figure 9b the proposed method. Results show that the positions of the identification data achieve better performance than [30]. When the attack strength is 20%, the anti-attack ability of our method exceeds 60% for three different cores. However, the method [30] shows a sharp decline when the attack strength exceeds 10%, showing the anti-attack ability of the proposed method to be encouraging and promising.

The anti-attack ability of the proposed scheme to the other three comparative schemes is also compared, as shown in Table 2. Xu et al. proposed a distributed data hiding scheme that cannot resist physical attacks, replay attacks, and machine learning attacks [29]. Cui et al. proposed a scheme that can resist replaying attacks although it can do nothing against physical attacks, machine learning attacks and the false attacks [30]. The scheme proposed by Long et al. has the ability against replay attacks and fake attacks, although it cannot resist physical attacks and machine learning attacks [28]. Finally, the proposed scheme has the ability against all former types of attacks.

### 4.4. Similarity Evaluation

In order to reflect the effect of identification data on the functionality of the IP circuit, the location conversion coefficient $HS_{ab}$ is utilized to evaluate the secrecy of identification data. This can be defined as the quadratic sum of the difference between the inserted and the extracted identification data, as defined in (20).

(20) $$HS_{ab}=\underset{\left(u,v,w\right)}{\min}H_{ab}\left(u,v,w\right)=\sum_{a=1}^{N}\sum_{b=1}^{N}{\left\|x_{a}-u_{a}\right\|}^{2}+{\left\|y_{b}-v_{b}\right\|}^{2}$$

At this point, (*u*,*v*) represents the LUT positions of the IP circuit and, respectively, m and n are the numbers of rows and columns of the position array. A larger value *HS_ab_* represents better security after inserting the identification data. Experiments show that it is difficult to analyze the difference between the original circuit and the marked circuit using the logic analyzer when *HS_ab_* is greater than 30 dB. In this work, we utilize the method by combining Hausdorff distance and normalization parameter to evaluate the similarity of the inserted and extracted data. The similarity is better when the normalization parameter is more significant. The normalized parameter $H_{nc}$ can be calculated by (21).

(21) $$H_{nc}=\frac{\sum_{x=0}^{m-1}\sum_{y=0}^{n-1}f_{1}\left(x,y\right)\times f_{2}\left(x,y\right)}{\sqrt{\sum_{x=0}^{m-1}\sum_{y=0}^{n-1}f_{1}\left(x,y\right)}\sqrt{\sum_{x=0}^{m-1}\sum_{y=0}^{n-1}f_{2}\left(x,y\right)}}$$

**Figure 10:**
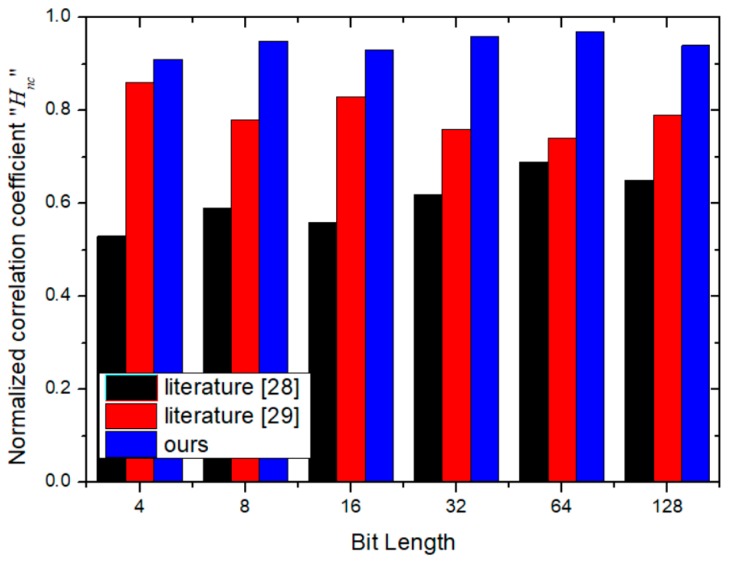
The evaluation of the normalized correlation coefficient.

**Figure 11:**
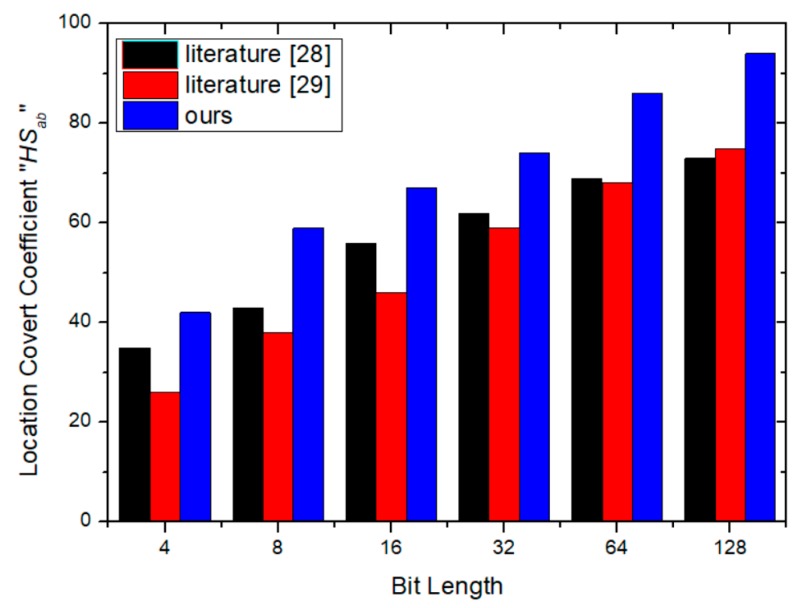
The evaluation of location covert coefficient.

In (21), *f_1_* and *f_2_* denote the inserted and the extracted identification data respectively, also *M* and *N* are respectively the bit lengths of both identification data. In the ideal case, the normalization parameter *H_nc_* = 1. As shown in Figure 10 and Figure 11, with the increase of the bit length, the *HS_ab_* and *H_nc_* are larger than that of the similar schemes, which demonstrates the excellent performance of the proposed scheme.

## 5. Conclusions and Future Work

With the rapid advances of highly sophisticated electronics and chips in IoT devices and increasing industrial and social applications, IP authentication attracts more attention to malicious attacks. Chips must be verified and secure protected as thoroughly as designs for any other applications in IoT environments, as the high volumes expected for several different types of IoT devices mean that any vulnerability to such chips would be enormously expensive. In recent years, several methods have been proposed for IP protection, although some methods are complicated in copyright authentication after suffering attacks. In this paper, the main contributions are twofold: (1) a position mapping algorithm based on Hausdorff distance is proposed to generate the virtual positions, used to determine the initial positions for inserting the identification data and storing it in the key file. During authentication, the virtual positions can be found through the key file and Hausdorff distance function. By matching the characteristic, the real positions of identification data can be found for authentication, and (2) a method to compute the Hausdorff distance is proposed. According to the position characteristic, an optimization model is created, where the maximum Hausdorff distance is selected as the unidirectional distance among LUT resources. The proposed method could transform the computation of Hausdorff distance into the minimum distance between two points. As a result, the efficiency and stability are improved, shown and validated the proposed method, which has good performance for secured insertion of identification data. 

Furthermore, the anti-attack ability is also enhanced. Even though some abecedarian researches on the Hausdorff distance-based IP authentication technique have been attempted, there are still some challenges. The proposed scheme mainly utilizes the distance as a measurement of similarity between two positions, and the position matching by the distance between LUTs should require the participation of the credible third party. Nevertheless, a full, credible third party may not exist in the industry community, although we assumed the existence of the credible third party. In this way, the IP authentication technique requires further investigation in practicability, security, and reliability.

## Figures and Tables

**Table 1:** Overhead evaluation for four types of intellectual property (IP) circuits.

IP Circuit	Occupied Resources	Algorithm	Average Hausdorff Distance	Time (ns)	ε
Audio	424	Literature [30]	-	12.77	0.423
		Literature [29]	-	10.56	0.439
		Literature [28]	-	16.56	0.487
		ours	14.43	7.64	0.439
DES	7064	Literature [30]	-	12.48	0.437
		Literature [29]	-	12.16	0.498
		Literature [28]	-	12.15	0.436
		ours	17.53	5.13	0.427
RS	7392	Literature [30]	-	13.57	0.473
		Literature [29]	-	12.49	0.416
		Literature [28]	-	13.67	0.435
		ours	13.42	6.84	0.484
Cache	14352	Literature [30]	-	12.29	0.469
		Literature [29]	-	14.3	0.468
		Literature [28]	-	17.82	0.479
		ours	15.74	5.87	0.467

**Table 2:** The comparison of the anti-attack ability.

Program	Replay Attack	Physical Attack	Machine Learning Attack	Fake Attacks
Xu	No	No	No	Yes
Cui	Yes	No	No	No
Long	Yes	No	No	Yes
Ours	Yes	Yes	Yes	Yes
